# One-time nitrogen fertilization shifts switchgrass soil microbiomes within a context of larger spatial and temporal variation

**DOI:** 10.1371/journal.pone.0211310

**Published:** 2019-06-18

**Authors:** Huaihai Chen, Zamin K. Yang, Dan Yip, Reese H. Morris, Steven J. Lebreux, Melissa A. Cregger, Dawn M. Klingeman, Dafeng Hui, Robert L. Hettich, Steven W. Wilhelm, Gangsheng Wang, Frank E. Löffler, Christopher W. Schadt

**Affiliations:** 1 Biosciences Division, Oak Ridge National Laboratory, Oak Ridge, Tennessee, United States of America; 2 Department of Biological Sciences, Tennessee State University, Nashville, Tennessee, United States of America; 3 Chemical Sciences Division, Oak Ridge National Laboratory, Oak Ridge, Tennessee, United States of America; 4 Department of Microbiology, University of Tennessee, Knoxville, Tennessee, United States of America; 5 Environmental Science Division, Oak Ridge National Laboratory, Oak Ridge, Tennessee, United States of America; 6 Institute for Environmental Genomics and Department of Microbiology & Plant Biology, University of Oklahoma, Norman, Oklahoma, United States of America; Wilfrid Laurier University, CANADA

## Abstract

Soil microbiome responses to short-term nitrogen (N) inputs remain uncertain when compared with previous research that has focused on long-term fertilization responses. Here, we examined soil bacterial/archaeal and fungal communities pre- and post-N fertilization in an 8 year-old switchgrass field, in which twenty-four plots received N fertilization at three levels (0, 100, and 200 kg N ha^-1^ as NH_4_NO_3_) for the first time since planting. Soils were collected at two depths, 0–5 and 5–15 cm, for DNA extraction and amplicon sequencing of 16S rRNA genes and ITS regions for assessment of microbial community composition. Baseline assessments prior to fertilization revealed no significant pre-existing divergence in either bacterial/archaeal or fungal communities across plots. The one-time N fertilizations increased switchgrass yields and tissue N content, and the added N was nearly completely removed from the soil of fertilized plots by the end of the growing season. Both bacterial/archaeal and fungal communities showed large spatial (by depth) and temporal variation (by season) within each plot, accounting for 17 and 12–22% of the variation as calculated from the Sq. root of PERMANOVA tests for bacterial/archaeal and fungal community composition, respectively. While N fertilization effects accounted for only ~4% of overall variation, some specific microbial groups, including the bacterial genus *Pseudonocardia* and the fungal genus *Archaeorhizomyces*, were notably repressed by fertilization at 200 kg N ha^-1^. Bacterial groups varied with both depth in the soil profile and time of sampling, while temporal variability shaped the fungal community more significantly than vertical heterogeneity in the soil. These results suggest that short-term effects of N fertilization are significant but subtle, and other sources of variation will need to be carefully accounted for study designs including multiple intra-annual sampling dates, rather than one-time “snapshot” analyses that are common in the literature. Continued analyses of these trends over time with fertilization and management are needed to understand how these effects may persist or change over time.

## Introduction

Cultivation of dedicated bioenergy crops is of interest to sustain long-term energy supplies [1]. The International Energy Agency predicts that biofuels could satisfy more than a quarter of world needs for transportation energy by 2050 [2]. Switchgrass (*Panicum virgatum* L.) has been a prominent candidate as an energy crop due to its high biomass yield, low maintenance and limited-input requirements [3, 4], and high adaptability to marginal sites [5]. Such characteristics may allow switchgrass crops to be used to reclaim degraded or abandoned agricultural lands while reserving fertile lands for food production [6]. With its well-developed and deep rooting systems, switchgrass may also improve belowground carbon storage and nutrient acquisition [7], and potentially modulate the diversity of below-ground and plant-associated microbiomes.

Soil microbial communities play fundamental roles in terrestrial ecosystems, such as regulating the decomposition of organic matter as well as driving nutrient cycles and energy flow [8, 9]. To this end, these soil microbiomes have considerable effects on soil quality and agricultural sustainability [10]. However, soil management with fertilizer additions may shift soil microbial abundances, composition, and functions by affecting soil physical and chemical characteristics [11] as well as perturbing plant-microbe interactions and symbioses [12]. For example, laboratory studies showed that N addition depresses soil microbial activity, microbial biomass, and enzyme activities by shifting the metabolic capabilities of soil bacterial communities toward the decomposition of more labile soil carbon pools [13]. In addition, nutrient inputs have been shown to shift the composition of soil microbial communities in consistent ways in grasslands across the globe with reduced average genome sizes of microbial community members following nutrient amendment, leading to decreased relative abundances of some important microbial functional groups, such as methanogenic archaea, oligotrophic bacteria and mycorrhizal fungi [14]. Several reasons may account for such microbial responses to fertilization. Long-term fertilization may cause soil acidification, and thus alter soil microbial diversity and composition [11, 15]. Additionally, nutrient amendments may have direct effects on organic matter decomposition, leading to changes in the quantity and quality of resources available for microbes, and therefore reshape microbial community structure based on their substrate utilization and allocation patterns [16]. However, our understanding of the mechanisms of N fertilization effects on microbial communities are mostly based on long-term fertilization experiments, in which potentially interacting edaphic soil properties are often significantly altered by soil management over time. Although transient nutrient enrichment effects upon terrestrial microbial C and N processes have been reported [17–19], our understanding of the immediate response of the below-ground microbial community to N inputs is still limited, and sometimes inconsistent with results of long-term experimental data [20–22]. Additional research is thus necessary to evaluate microbial community dynamics and their interactions with nutrient cycling under the no-, low- or periodic fertilization regimes that would be optimal for sustainable perennial bioenergy crop production scenarios [23, 24].

Besides soil nutrient availability, soil microbial distributions are influenced by a wide range of soil characteristics, such as soil pH, substrate quantity and quality, moisture and oxygen levels, nearly all of which vary with soil depth [25] and over seasons [26]. Soil depth and sampling time in a growing season thus influence patterns of spatial and temporal community variation [27, 28]. Compared to top soils, subsurface soils have higher mineral content, less aeration, and lower organic carbon availability. Thus, microbial biomass and diversity typically decrease rapidly with depth in the soil profile [29]. Often, most variability in microbial community composition occurs in surface soils, while deeper soils have more similar communities regardless of soil management [25]. Seasonal variability also has a large influences on microbial communities [30]. For example, seasonal changes in temperature and soil moisture can directly shape microbial communities [31, 32]. Moreover, seasonal changes in plant growth and allocation can indirectly affect microbial communities through changes in soil C inputs [33, 34]. Lauber et al. [26] investigated the temporal variability of bacterial communities in different ecosystems, showing that most of the temporal variation in bacterial composition within an agricultural field could be explained by soil moisture and temperature variation. Given these previous studies, it is possible that the shifting spatial and temporal patterns of soil microbial communities may overwhelm short-term soil nitrogen management effects and needs to be accounted for in such assessments.

Here, we used high-throughput barcoded amplicon sequencing of ribosomal RNA marker genes to assess short-term effects of one-time N fertilization on the spatio-temporal variation of soil microbial communities in an 8 year-old switchgrass field, over two soil depths and across four sampling seasons. We hypothesized that (1) one-time nutrient inputs could significantly change above-ground plant yields and substrate quality, and thus re-shape soil bacterial and fungal communities, but that short term N effects would be modest compared to existing spatio-temporal variation, (2) bacterial and fungal composition would differ spatially and temporally, but the response of these communities to the N-fertilization would be taxon specific.

## Materials and methods

### Site characterization, experimental design and plant and soil sampling

**Fig 1:**
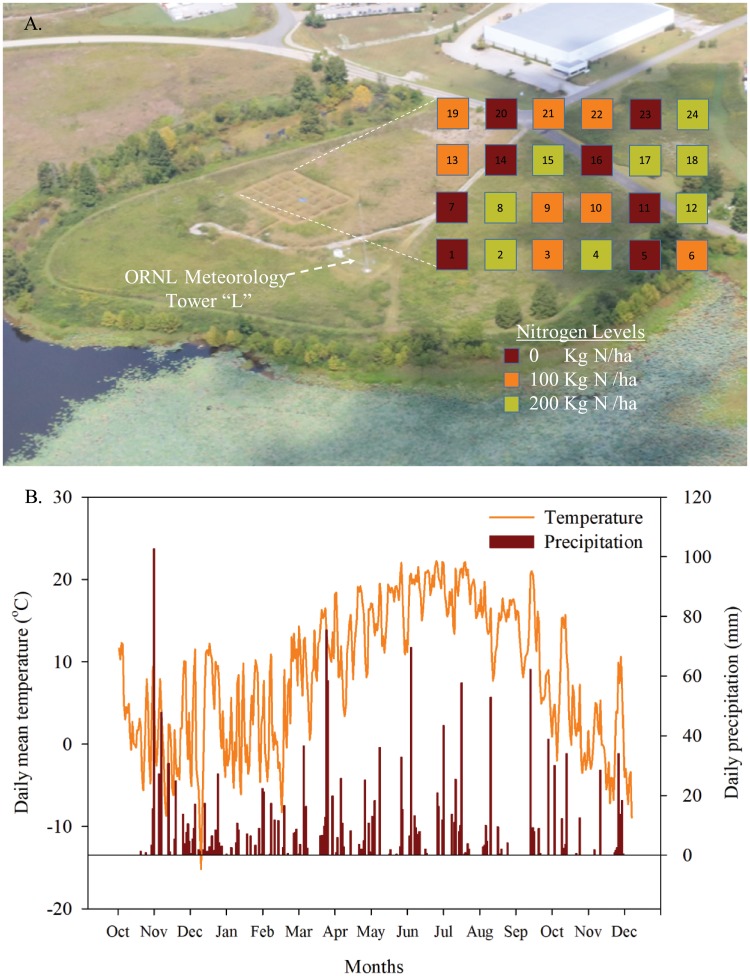
A. An ariel photograph from August 2018 showing the layout of twenty four plots (5 m × 5 m) with three N fertilization levels (0, 100, and 200 kg N ha^-1^) and eight replicates based on a complete randomized design. B. Daily mean temperature and precipitation (November 2016 to December 2017) from the adjacent meteorological tower in Oak Ridge, Tennessee, USA.

The experiment was established in an eight year-old switchgrass field near the Heritage Center, located on US DOE land, in Oak Ridge, Tennessee, USA (35.9255 N, 84.3947 W) adjacent to the ORNL Meteorology Network tower “L” with a 25+ year record of data available online (http://metweb.ornl.gov—Fig 1). The 10-year mean annual temperature and annual precipitation at the site were 14.1°C and 1436 mm, respectively. The field was in pasture and hay rotations when taken over by US Army in the 1940s during the Manhattan Project. However, due to proximity of floodplain of the Clinch River and Poplar Creek, the land was never developed and instead maintained as wildlife habitat and riparian buffer in a field of mixed grasses and forbs, using a combination of mowing and prescribed burning for management. In 2009, under contract for UT Institute of Agriculture and Genera Energy, the site was cleared, seeded with an ‘Alamo’ switchgrass variety, and subsequently managed for switchgrass production. After the initial contract expired in 2012 when Genera Energy changed the focus of their local efforts, the site has remained in switchgrass, but has again been managed as buffer and wildlife habitat and maintained only with periodic prescribed fire and mowing. In mid-December 2016, the switchgrass field was mowed to a 10-cm stubble height and twenty four plots (5 m × 5 m) were set up including three N fertilization levels (0, 100, and 200 kg N ha^-1^) with eight replicates based on a complete randomized design (Fig 1). A 2.5-m inter-plot “alley way” is periodically mowed to allow access and separate the plots between treatments and replicates (Fig 1). Just before spring emergence of the switchgrass (March 30, 2017) commercial ammonium nitrate (34% nitrogen) was hand-applied to fertilizer-treatment plots with N fertilization levels of 100, and 200 kg N ha^-1^. Post-emergence (June 20, 2017), all plots were treated with Garlon 3A herbicide as prescribed by the manufacturer to help control broadleaf weeds. After fall senescence (November 13, 2017) above-ground biomass of switchgrass was measured using a sickledrat to harvest all aboveground biomass from a 0.1 m^2^ area at four randomly chosen locations in each plot [33]. The four samples of aboveground biomass were pooled by plot in paper bags, oven dried at 70°C, and weighed to determine dry mass per unit area. A subsample of the plant material was then ground into powder using a laboratory mill before total C and N were determined by dry combustion method using a Perkin-Elmer 2400 CHN analyzer (Perkin-Elmer Corporation, Norwalk, CT, USA).

For soil DNA and chemical characterization, four sets of soil samples were collected across seasons including Winter 2016 (December 16, 2016 –Pre-fertilization), Spring 2017 (April 5, 2017 –Post-fertilization/emergence), Summer 2017 (July 5, 2017 –Mid-growing season), and Late Fall 2017 (November 15, 2017—Senescence). At each sampling event, soil cores (2.5 cm diameter ×15 cm height) were collected randomly from each plot and separated into two depth increments of 0–5 and 5–15 cm as preliminary tests had showed that soil of 0–5 cm had greater amount of soil DNA than 5–15 cm, and that soil sampling of depths below 15 cm were inconsistent across plots due to a rocky layer underlying the plots in the southwest corner of the experiment. Soils collected in Winter 2016 (before N fertilization) were used to optimize soil methods for microbiomes and metagenomes assessments, and assess whether there were any systematic pre-existing differences across plots in the experiment. Soils collected following N fertilization, *i*.*e*., Spring 2017, Summer 2017, and Late fall 2017, were used to compare microbial communities among N treatments. Soil collected for all four seasonal sampling dates were used to assess how soil depth and sampling season affected microbial communities across all three nitrogen treatments. All soil samples for microbial analyses were transported on dry ice to the lab and stored at -80° C prior to soil DNA extraction. Total C and N were determined on samples collected in the Summer of 2017 using the dry combustion method and soil inorganic N (NH_4_^+^-N and NO_3_^-^-N) was analyzed using a FIA QuikChem 8000 autoanalyzer (Lachat Instruments, Loveland, CO, USA). Generally, 0–5 cm soils had an average soil pH of 5.8, 4.5% total soil C, 0.2% total soil N, 10.2 mg kg^-1^ NH_4_^+^, and 1.3 mg kg^-1^ NO_3_^-^, while 5–15 cm soil had a significantly lower average soil pH of 5.2, 1.8% total soil C, 0.03% total soil N, 1.8 mg kg^-1^ NH_4_^+^, and 0.3 mg kg^-1^ NO_3_^-^.

### DNA extraction, rRNA gene amplicon sequencing, and metagenomic sequencing

Approximately 10 g of soil from each sample was homogenized in a mortar and pestle with liquid N_2_, and soil DNA was extracted from a 0.25 g aliquot of the soil sample using the MoBio DNeasy PowerSoil Kit (Qiagen, Carlsbad, CA, USA) according to the manufacturer’s instructions. DNA concentrations were determined and purity was confirmed by the ratio of absorbance at 260 and 280 nm (1.70–1.90) using a NanoDrop 1000 spectrophotometer (NanoDrop Products, Wilmington, DE, USA).

A two-step PCR approach was used to barcode tag templates with frameshifting nucleotide primers for amplicon sequencing [35] with some modifications previously described [36]. To increase phylogenetic coverage for community analysis of bacteria, archaea, and fungi, a group of nine forward and six reverse primers for bacteria and archaea, and another group of eleven forward and seven reverse primers for fungi, mixed at equal concentration of 0.5 μM were used to target 16S rRNA V4 region and fungal ITS2 rRNA, respectively [36]. Primary PCR was conducted for 5 cycles of 1 min at 95 °C, 2 min at 50 °C, and 1 min at 72 °C, followed by a final elongation of 5 min at 72 °C. This PCR product was then cleaned up using Agencourt AMPure beads (Agencourt Bioscience, Beverly, MA, USA) and eluted in 21 μL of nuclease-free water. To tag amplicons with barcoded reverse primers and forward primers, 20 μL of purified DNA fragments from the primary PCRs were added to 50 μL secondary PCR assays, which were initiated at 95 °C for 45 sec, followed by 32 cycles of 15 sec at 95 °C, 30 sec at 60 °C, and 30 sec at 72 °C, followed by a final elongation of 30 sec at 72 °C. The use of separate tagging reactions can help reduce heterodimers because PCR clean-up more efficiently removes shorter primers [35]. Up to 96 secondary PCR products were then pooled based on agarose gel band intensity, followed by a second clean-up with Agencourt AMPure beads (Agencourt Bioscience, Beverly, MA, USA) using 0.7–1 of bead-to-DNA ratios. The mixtures of the purified 16S rRNA gene or ITS amplicon fragments were then paired-end sequenced on Illumina MiSeq platform (250×2 paired end, v2 chemistry) (Illumina, San Diego, CA, USA) using a 9 pM amplicon concentration. For all amplicon sequencing data, any sample with fewer sequence reads than 10000 for 16S and 5000 for ITS, respectively, was removed and re-sequenced to ensure the minimum sequencing depths.

To examine microbial community potential and function prior at the site and make sure that there were no preexisting differences in either microbial community or functional profiles across N plots prior to N fertilization, DNA extracted from soil samples collected in Winter 2016 were pooled to form four composite samples, and metagenomic sequence data were generated and compared to the results of functional predictions by PICRUSt (Phylogenetic Investigation of Communities by Reconstruction of Unobserved States) [37]. Specifically, for each of two soil depths, DNA from No. 1–12 and 13–24 plots was pooled together, respectively (two depths × two replicates). Shotgun metagenomes were prepared using Nextera XT sequencing libraries (Illumina, San Diego, CA) according to the manufacture’s recommendations using 500ng of DNA (15031942 v03). Final libraries were validated on an Agilent Bioanalyzer (Agilent, Santa Clara, CA) using a DNA7500 chip and concentration was determined on an Invitrogen Qubit (Waltham, MA) with the broad range double stranded DNA assay. Barcoded libraries were pooled and prepared for sequencing following the manufactures recommended protocol (15039740v09, Standard Normalization). One paired end sequencing run (2 x 300) was completed on an Illumina MiSeq instrument (Illumina, San Diego, CA) using v3 chemistry. The sequencing depths obtained were similar among four samples (10,069,906–11,652,583 reads per sample).

### Bioinformatic and statistical analyses

Forward and reverse primers were trimmed with Cutadapt [38]. Paired-end sequencing data were then joined and demultiplexed using QIIME [39] with quality filter at Phred > 19. Chimeras of trimmed and filtered sequences were identified and removed using a usearch method in QIIME. Operational taxonomic units (OTUs) with 97% identity were picked with the open reference algorithm and usearch61 otu-picking method. Taxonomy was assigned using the RDP (Ribosomal Database Project) taxonomy-assignment method [40] against the most recent version of Greengenes database (13.8) for 16S rRNA sequencing data and UNITE database (12.11) for ITS sequencing data. All global singletons were removed from the dataset. The 16S and ITS OTUs were further analyzed for alpha and beta diversity using QIIME. For alpha diversity analysis, all sequences were rarefied to the depths of 10000 for 16S and 5000 for ITS, respectively. Bray-Curtis distance metrics were applied for analyzing beta diversity. The beta diversity of both amplicon and metagenomic sequencing was based on relative abundance, which can only be related to the community structure and potential functions, but cannot be directly interpreted to ecosystem functions. To test whether bacterial community functional traits could be accurately predicted from 16S rRNA genes, we used PICRUSt [37] for comparison with 4 metagenome libraries described below. The MiSeq sequences were deposited on NCBI Sequence Read Archive (SRA) database under the BioProject accession number of PRJNA512218.

Soil metagenome sequences were uploaded to Rapid Annotation using Subsystems Technology for Metagenomes (MG-RAST; http://metagenomics.anl.gov) [41] under project accession number mgp22000, and annotated using the RefSeq database for taxonomic assignment and the SEED Subsystems database for functional classification (maximum e-value cutoff was 1e^-5^, minimum identity cutoff was 60%, and minimum alignment length was 50).

One-way analysis of variance (ANOVA) of a completely randomized design (SAS 9.3, SAS Institute Inc. Cary, NC, USA) was used to assess significant differences in above-ground yields and plant C/N contents among N fertilization levels. A three-factor ANOVA of a completely randomized design was used to analyze microbial alpha diversity and the abundances of microbial taxonomic groups among the three N fertilization levels, two soil depths, and four seasonal sampling dates. Microbial beta diversity was compared using a three-factor PEMANOVA method (N fertilization levels, soil depths, and sampling season) with 9999 permutations conducted in PRIMER (Plymouth Routines in Multivariate Ecological Research Statistical Software, v7.0.13, PRIMER-E Ltd, UK). A RELATE analysis was also performed to evaluate the relatedness between bacterial and fungal beta diversity by calculating a Spearman’s Rho correlation coefficient in PRIMER. The DistLM (distance-based linear model) function in PRIMER was used to evaluate the associations of above-ground yields and plant C/N contents with bacterial and fungal beta diversity [42]. Heat maps were constructed using HeatMapper [43] to represent all taxonomic groups at genus level that differed significantly (*P* < 0.05) among three N fertilization levels, two soil depths, and four sampling dates. Venn’s diagrams were also constructed to visualize how many significantly affected bacterial/archaeal and fungal genera were shared between the factors of soil depth and sampling date using Venny 2.1.0 [44]. Additionally, Pearson’s correlation coefficients were examined to further evaluate relationships between the relative abundances of taxa and N fertilization rates.

## Results

### Spatial variation in microbial community structure and function pre-nitrogen addition

**Fig 2:**
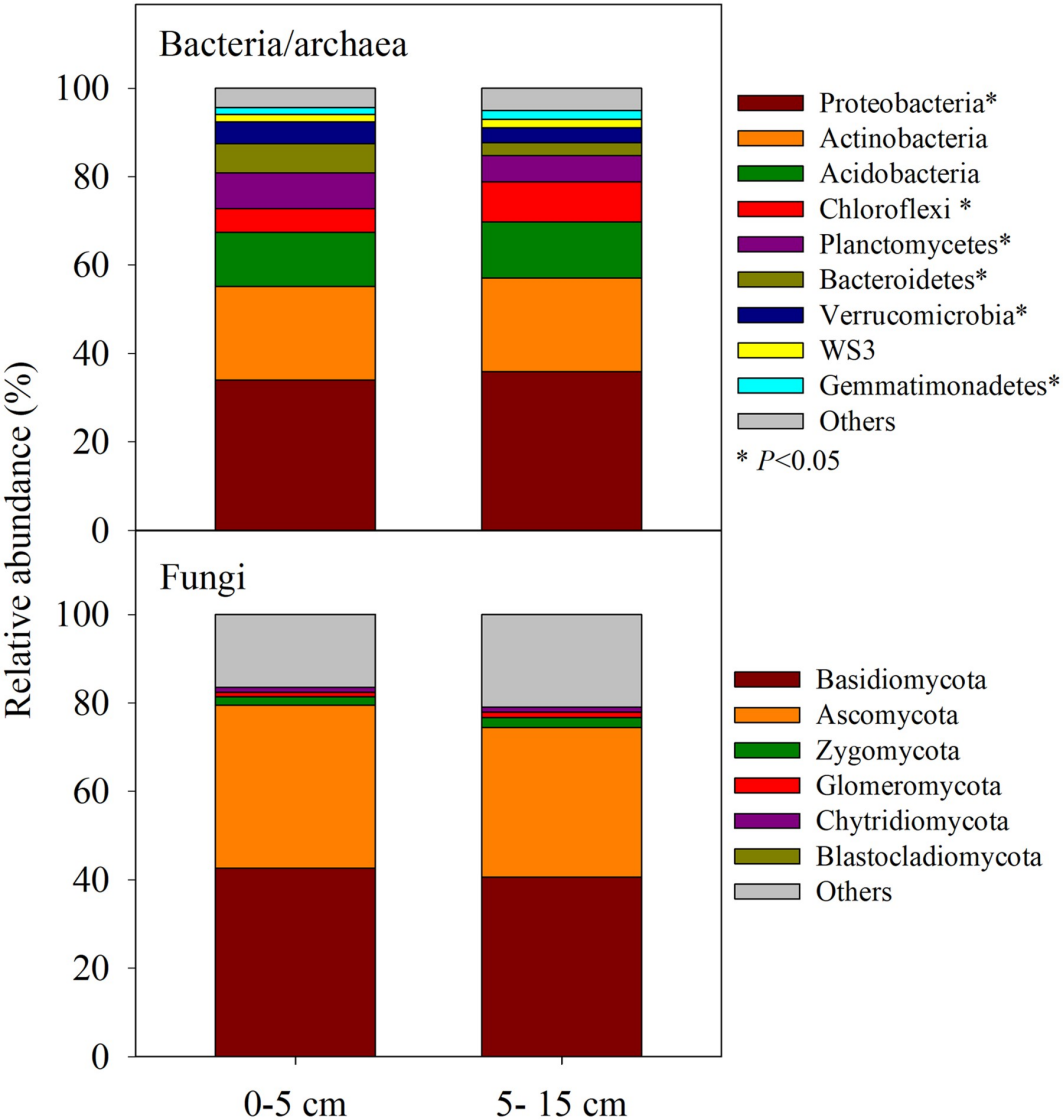
Relative abundances of bacterial/archaeal and fungal dominant phyla (average abundances > 1%) affected by two soil depths (0–5 and 5–15 cm). Asterisks indicate significant difference at α = 0.05 between two soil depths.

Both 16S rRNA gene and ITS region amplicon sequencing revealed no significant pre-existing differences in alpha or beta diversity in either the bacterial/archaeal or fungal communities across the 24 plots in this switchgrass field before N fertilization, however diverse bacterial/archaeal and fungal taxa were observed (Fig 2). Bacterial communities varied significantly by depth (*P* < 0.05) with the 0–5 cm soil layer having greater Planctomycetes (8%), Bacteroidetes (7%), and Verrucomicrobia (5%), but less abundance of Proteobacteria (34%), Chloroflexi (5%), and Gemmatimonadetes (2%) than the deeper layers (Fig 2). Surprisingly, fungal phyla level analyses did not show any differences between the soil depths examined in these switchgrass soils.

Shotgun metagenomes also showed high taxonomic and functional diversity in the switchgrass field (S1 Fig). However, when phylogenetic assignments of the metagenome reads were compared to the relative abundance in 16S rRNA gene amplicon analyses, soil metagenomes indicated significant differences in the datasets across several of the dominant phyla. For example, there was a 45% increase in Proteobacteria, a 15-fold increase in Firmicutes, and a 2-fold increase in Cyanobacteria in the shotgun metagenomes when compared to 16S rRNA gene amplicon analyses. Other phyla, such as Acidobacteria, Planctomycetes, and Chloroflexi, were reduced by as much as 52–63% in soil metagenomes when compared to 16S rRNA gene amplicon analyses from the same samples and dates. Soil metagenome predicted functional gene profiles were compared to those predicted from PICRUST-based analysis of 16S rRNA gene amplicon data and also indicated significantly different profiles (S2 and S3 Figs). As metagenome-based analyses of all replicate samples on all dates was impractical, and PICRUST-based analyses of seasonal functional gene patterns and responses to fertilization were not consistent with metagenome analyses, these approaches were not pursued further for this study.

### Microbial alpha and beta diversity post-nitrogen addition

**Fig 3:**
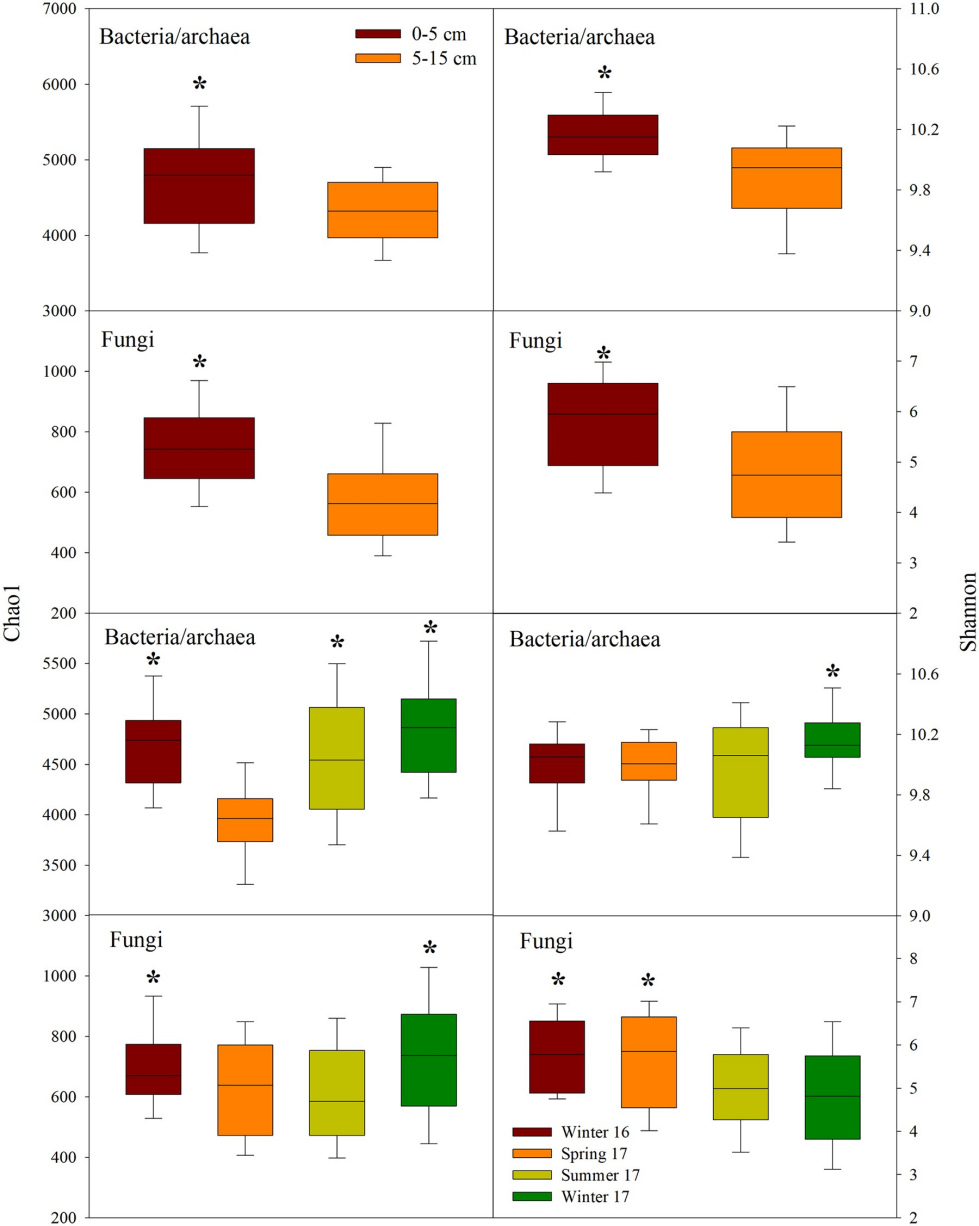
Box plots showing Chao1 richness and Shannon diversity of bacterial/archaeal and fungal communities affected by two soil depths (0–5 and 5–15 cm) and four sampling seasons (Winter 2016, Spring, Summer, and Fall 2017). Sequence depths were 10000 for 16S and 5000 for ITS. Asterisks indicate significant difference at α = 0.05 between two soil depths or among four sampling seasons.

Although neither bacterial/archaeal nor the fungal alpha diversity were significantly affected by N fertilization levels, both the community richness (Chao1 index) and diversity (Shannon index) showed significant spatio-temporal changes (*P* < 0.05) (Fig 3). Between the two soil depths, the 0–5 cm layer had significantly higher Chao1 richness and Shannon evenness indices in both the bacterial/archaeal and fungal communities compared to the 5–15 cm layer (*P* < 0.05). In analyses of seasonal variation, Chao1 diversity showed a similar pattern. Spring 2017 had lower richness in the bacterial/archaeal community, while Winter 2016 and Fall 2017 had significantly greater richness in fungal communities (*P* < 0.05). Shannon diversity indices showed significant divergence across seasons (*P* < 0.05), and the bacterial/archaeal community was more evenly distributed in Fall 2017, whereas the fungal community was more uneven in Summer and Fall 2017 than the other two sampling seasons (*P* < 0.05).

**Fig 4:**
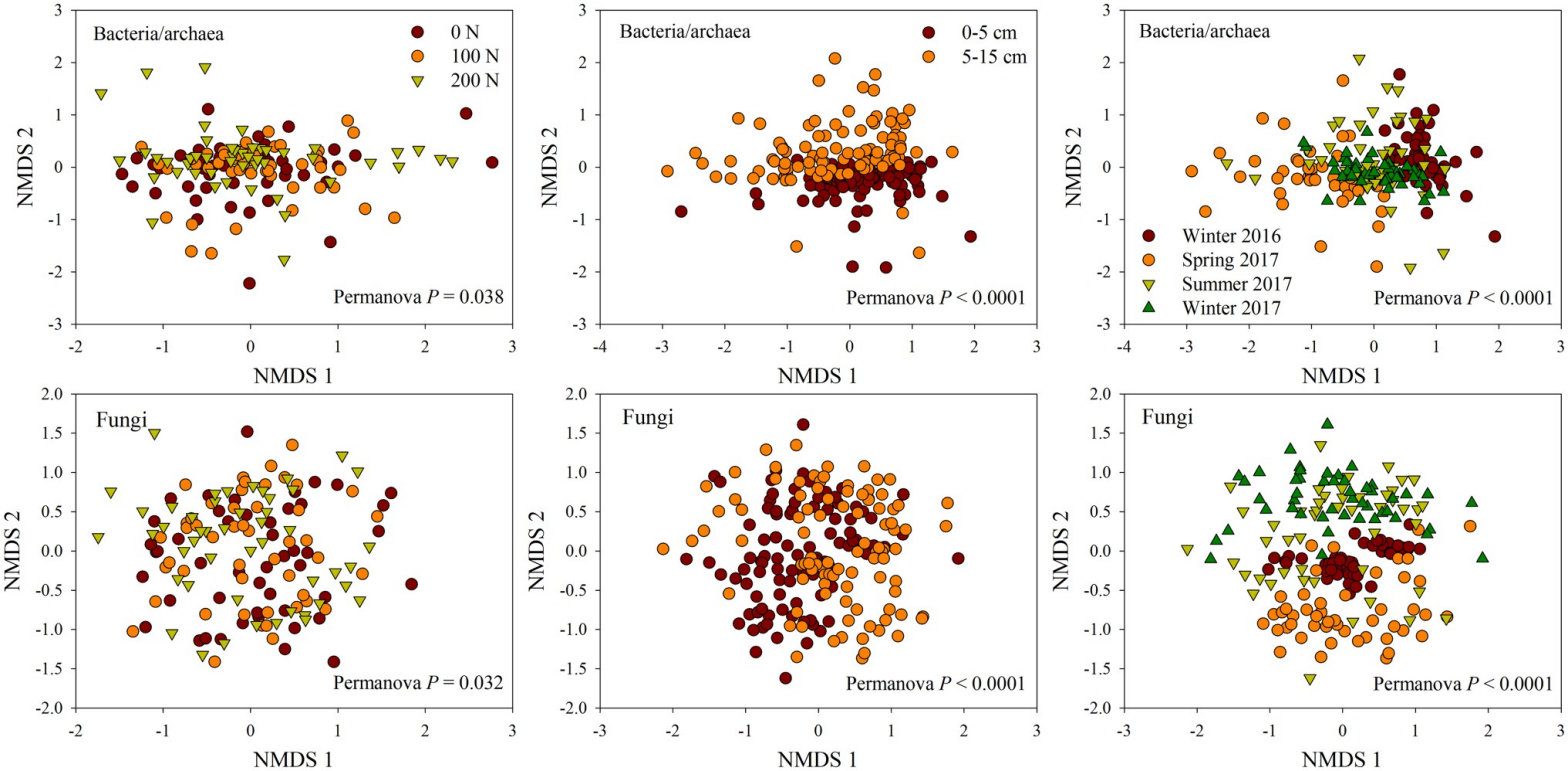
Non-metric multidimensional scaling (NMDS) analysis of bacterial/archaeal and fungal communities affected by three N fertilization levels (0, 100, and 200 kg N ha^-1^), two soil depth (0–5 and 5–15 cm), and four sampling seasons (Winter 2016, Spring, Summer, and Fall 2017). PERMANOVA *P* values were also given.

PERMANOVA tests showed that short-term application of N fertilizers could explain a small but significant amount of the variation in bacterial/archaeal and fungal community composition (*P* < 0.05) (Table 1 and Fig 4). Together, N fertilization effects could explain 3.4% of variation in bacterial/archaeal and 4.4% of fungal community variation calculated from the Sq. root of the PERMANOVA analyses (Table 1). However, the spatio-temporal variation (depth and season) were more significant than N effects for bacterial, archaeal and fungal communities (*P* < 0.0001) (Table 1 and Fig 4). Soil depth and sampling season explained approximately 16.8 and 17.3% of bacterial/archaeal community variation, respectively, and 12.4 and 22.4% of fungal community change, respectively (Table 1), thus indicating relatively small short-term effects of N fertilization on microbial communities when compared to the spatio-temporal variation. In addition, RELATE analyses further confirmed that bacterial/archaeal community structures were significantly related to the fungal community (Rho = 0.218, *P* < 0.01), suggesting that the patterns of spatio-temporal variation were generally similar in both bacterial/archaeal and fungal community distributions among tested plots and seasons.

**Table 1:** Three-factor PERMANOVA results for differences in bacterial/archaeal and fungal community structure affected by three N fertilization levels (0, 100, and 200 kg N ha^-1^), two soil depths (0–5 and 5–15 cm), and three sampling seasons following N input (Spring, Summer, and Fall 2017).

Source	df	SS	MS	PS-F	*P*(perm)	UP	Estimate	Sq.root
**Bacteria/Archaea**								
N	2	4010	2005	1.44	0.0384	9873	12.9	3.6
Depth	1	21742	21742	15.68	0.0001	9886	282.7	16.8
Season	2	31772	15886	11.46	0.0001	9884	302.1	17.3
N×Depth	2	1825	913	0.66	0.9879	9869	-19.7	-4.4
N×Season	4	5071	1268	0.91	0.6992	9809	-7.4	-2.7
Depth×Season	2	9614	4807	3.47	0.0001	9793	142.5	11.9
N×Depth×Season	4	3729	932	0.67	0.9995	9805	-56.8	-7.5
Res	126	1.8E+05	1386					
Total	143	2.5E+05						
**Fungi**								
N	2	8995	4497	1.29	0.0319	9808	21.5	4.6
Depth	1	14453	14453	4.17	0.0001	9859	152.6	12.4
Season	2	54894	27447	7.92	0.0001	9808	499.6	22.4
N×Depth	2	3897	1949	0.56	1.0000	9803	-63.2	-8.0
N×Season	4	14744	3686	1.06	0.3351	9720	13.8	3.7
Depth×Season	2	19295	9647	2.78	0.0001	9793	257.6	16.1
N×Depth×Season	4	9534	2383	0.69	1.0000	9704	-135.2	-11.6
Res	126	4.4E+05	3465					
Total	143	5.6E+05						

df, degrees of freedom; SS, sum of squares; MS, mean squares; PS‐F, pseudo‐F value; P(perm), permutation P-value based on 9999 permutations; UP, unique values of test statistic obtained under permutation; Estimate, estimated component of variation; Sq.root, square root of the estimated component of variation.

### Microbial taxonomic composition post-nitrogen addition

**Fig 5:**
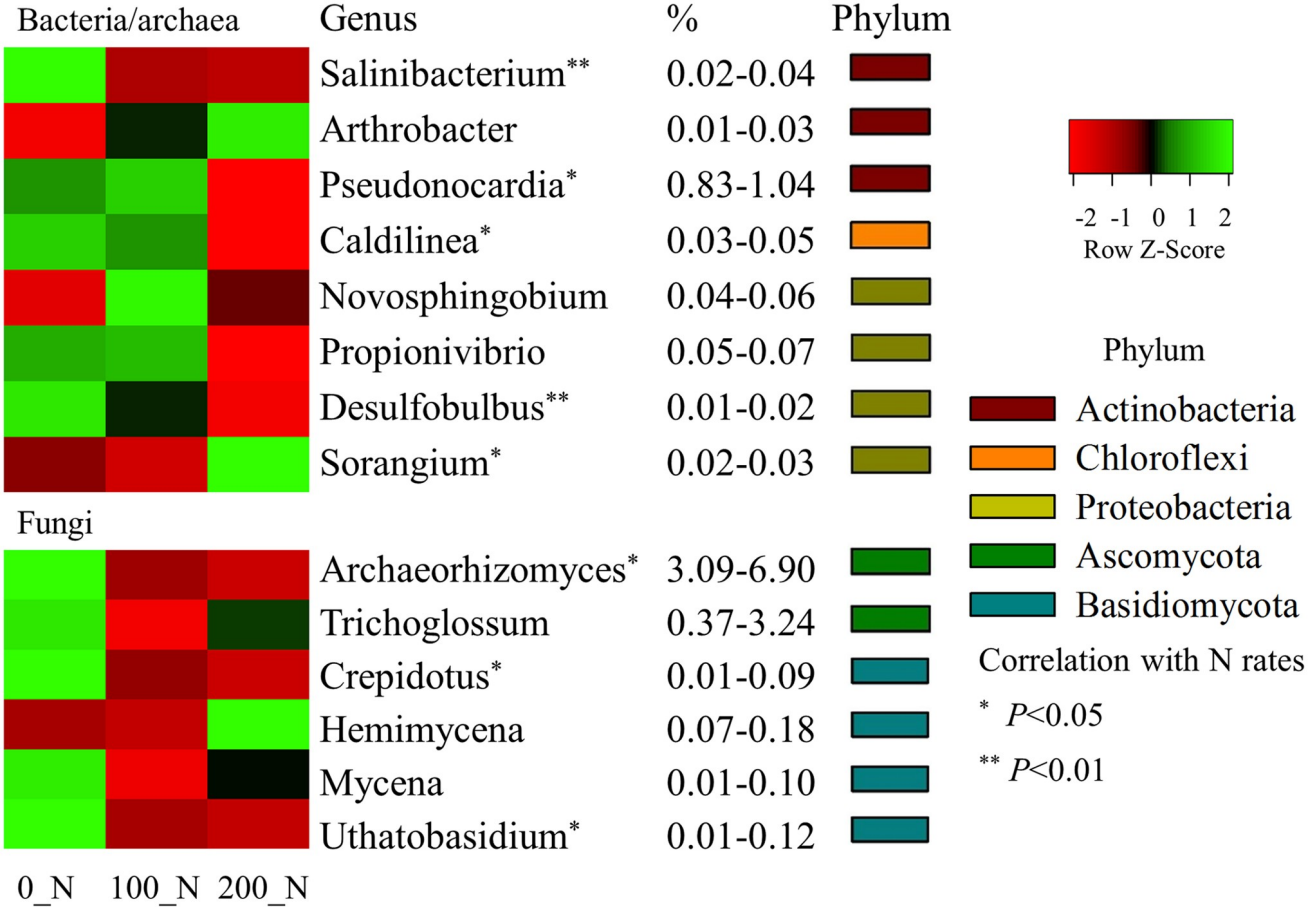
A heat map of relative abundance of bacterial/archaeal and fungal dominant taxonomic groups at genus level (average abundances > 0.1%) that were significantly affected by three N fertilization levels (0, 100, and 200 kg N ha^-1^). Asterisks indicate significant Spearman correlations of taxonomic abundance with N fertilization levels at α = 0.05.

Because N addition levels had no interaction with soil depth and sampling season (Table 1), N effects on microbial phylogenetic composition were assessed across both sampling depths and seasons (Fig 5). Generally, N fertilization caused significant differences in the recovered genus level composition for prominent members of the bacterial/archaeal (6%) and the fungal (5%) communities, respectively (relative abundance > 0.01%) (Fig 5). Specifically, for bacterial/archaeal community composition, N inputs at 200 kg N ha^-1^ were associated with significantly reduced relative abundances of *Salinibacterium* and *Pseudonocardia* (Actinobacteria), *Caldilinear* (Chloroflexi), and *Desulfobulbus* (Proteobacteria), but increased *Sorangium* (Proteobacteria) (*P* < 0.05) abundance, indicating that these taxonomic groups were significantly altered by the addition of synthetic N fertilizers. In the fungal community profiles, the application of N fertilizers at either 100 or 200 kg N ha^-1^ significantly decreased the proportion of *Archaeorhizomyces* (Ascomycota), as well as *Crepidotus* and *Uthatobasidium* (Basidiomycota) (*P* < 0.05).

**Fig 6:**
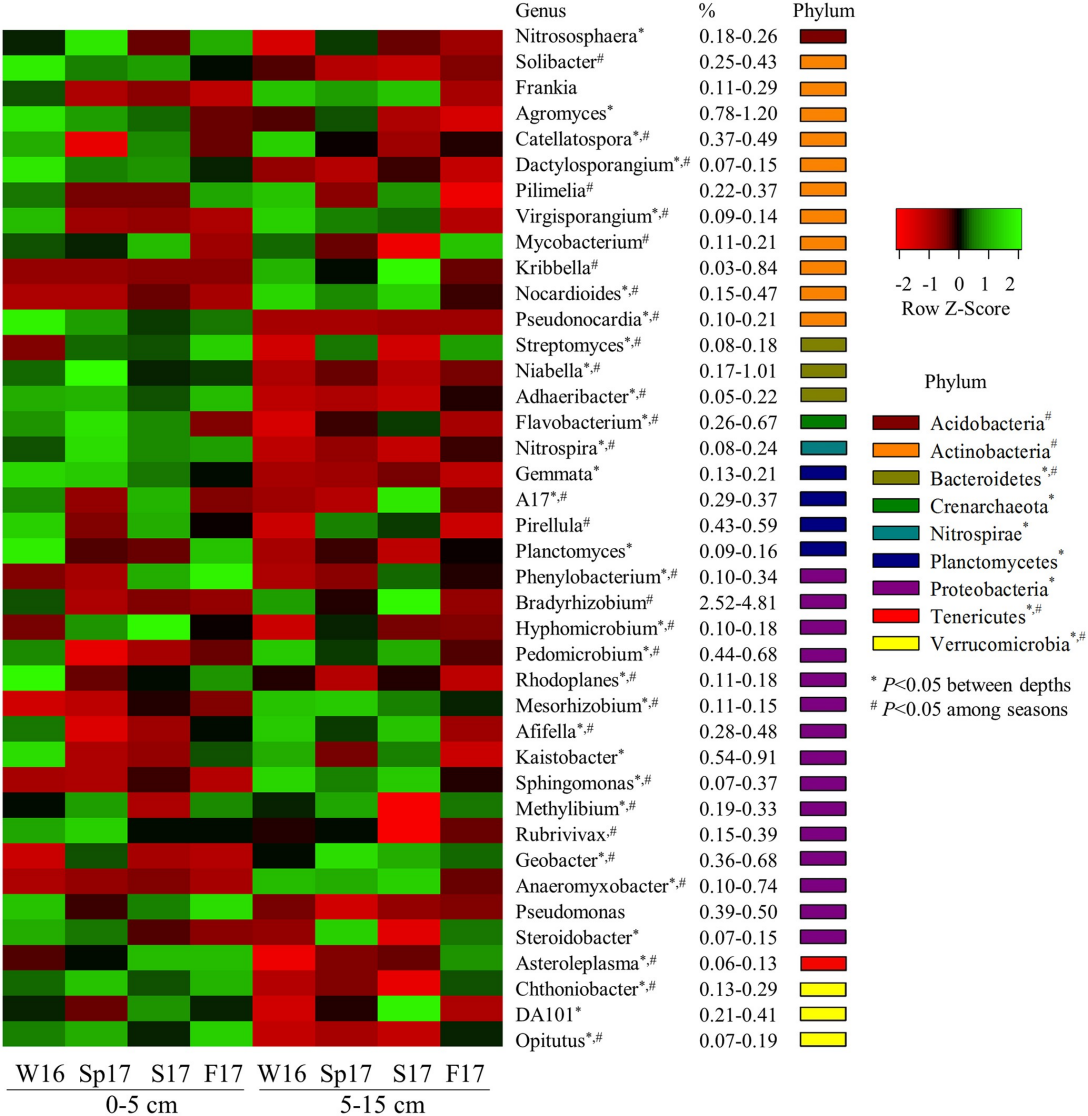
A heat map of relative abundance of bacterial/archaeal dominant taxonomic groups at genus level (average abundances > 0.1%) that were significantly variable between two soil depths (0–5 and 5–15 cm) and over four sampling seasons (Winter 2016, Spring, Summer, and Fall 2017). Asterisks indicate significant difference between two soil depths (0–5 and 5–15 cm) at α = 0.05. Number signs indicate significant difference over four sampling seasons (Winter 2016, Spring, Summer, and Fall 2017) at α = 0.05.

Both soil depth and sampling season resulted in more significant alteration to bacterial/archaeal community composition than N applications (Fig 6). For example, 81% bacterial taxonomic groups at the genus level (with relative abundance > 0.1%) differed significantly between 0–5 and 5–15 cm of soil layers (*P* < 0.05), and significant variation occurred even at the phylum level. Generally, the 0–5 cm soil layer had a greater abundance of the phyla of Bacteroidetes, Planctomycetes, and Verrucomicrobia, whereas the phyla Chloroflexi, Nitrospirae, and Proteobacteria dominated the 5–15 cm soil layer (*P* < 0.05) (Fig 6). Sampling season also caused significant changes in bacterial community composition with ~80% of bacterial genera significantly altered (*P* < 0.05) (Fig 6), mostly in the prominent phyla of Acidobacteria, Actinobacteria, Bacteroidetes, and Verrucomicrobia, suggesting that these taxonomic groups were most responsive to temporal changes.

**Fig 7:**
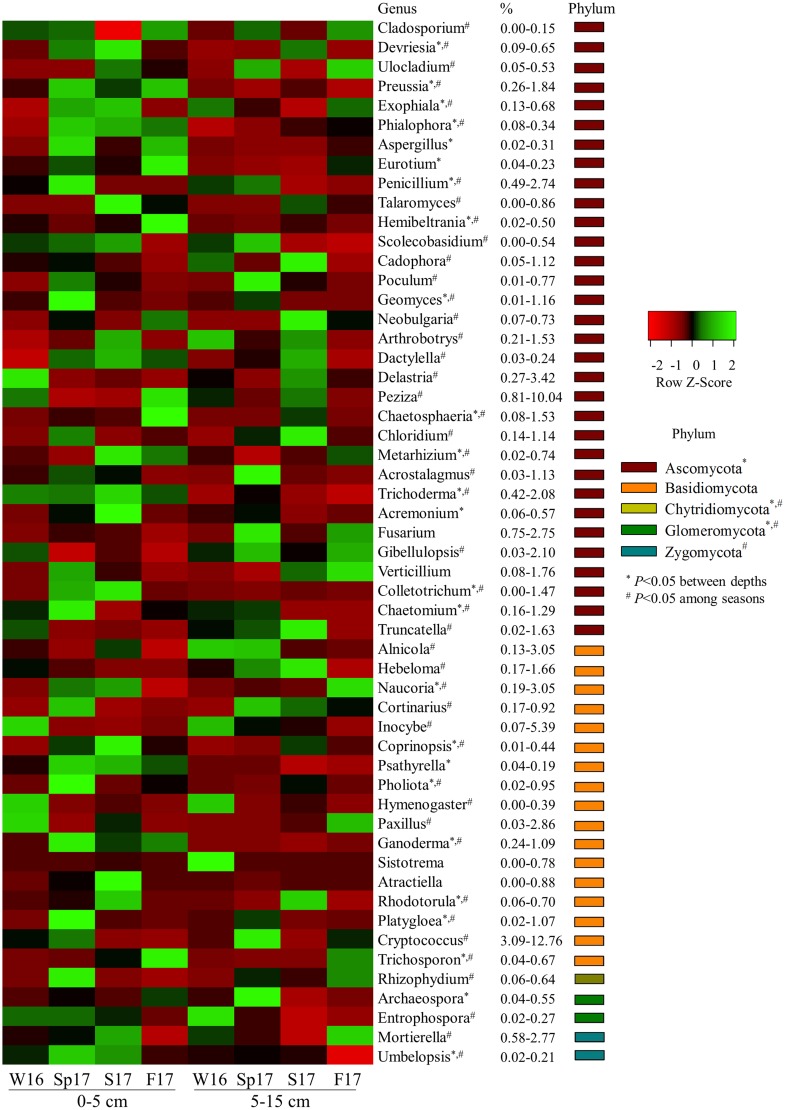
A heat map of relative abundance of fungal dominant taxonomic groups at genus level (average abundances > 0.1%) that were significantly variable between two soil depths (0–5 and 5–15 cm) and over four sampling seasons (Winter 2016, Spring, Summer, and Fall 2017). Asterisks indicate significant difference between two soil depths (0–5 and 5–15 cm) at α = 0.05. Number signs indicate significant difference over four sampling seasons (Winter 2016, Spring, Summer, and Fall 2017) at α = 0.05.

In the fungal community, only 54% prominent genera (of relative abundance >0.1%) showed significant changes between two soil depths, in which members of the phyla of Ascomycota, Chytridiomycota, and Glomeromycota were more prevalent in upper soil layer of 0–5 cm (*P* < 0.05) (Fig 7). Approximately 90% of the prominent fungal taxonomic groups classified at the genus level (relative abundance > 0.1%) also significantly varied over sampling seasons (*P* < 0.05) (Fig 7).

**Fig 8:**
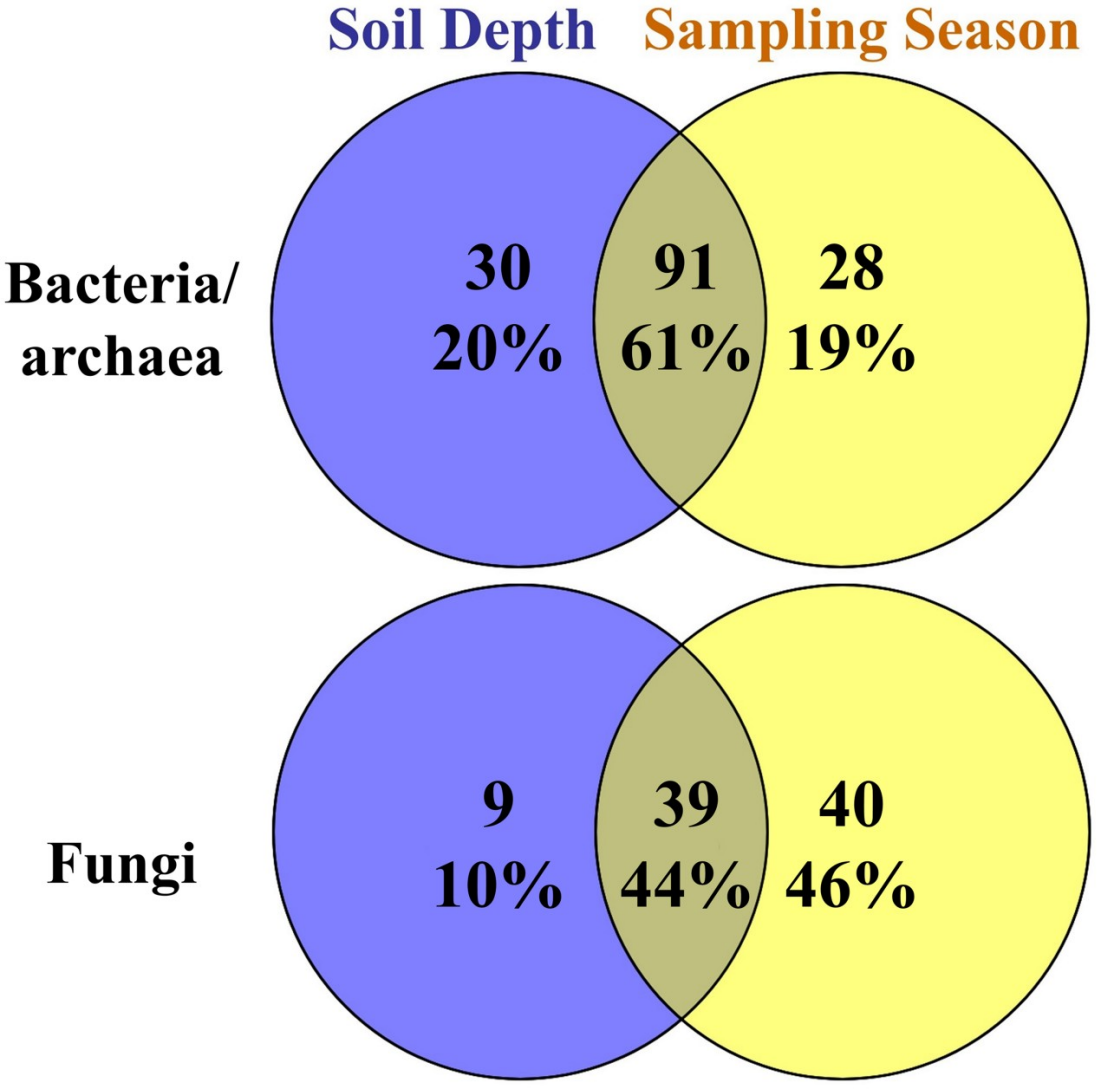
Venn’s diagrams showing significantly affected bacterial/archaeal and fungal dominant taxonomic groups at genus level (average abundances > 0.1%) shared between the factors of soil depth and sampling season.

Venn diagrams were used to better visualize these changes of bacterial/archaeal and fungal taxonomic groups affected by soil depth and sampling season (Fig 8). In bacteria, 61% of significantly affected genera were shared by the two factors of soil depth and sampling season, illustrating that most bacterial groups that differed between depths also responded to temporal change. In fungi, many more fungal taxonomic groups significantly varied across the four seasonal samples than with the soil depth (Fig 8), again indicating that temporal variation affected fungal community composition more significantly than spatial variation.

### Microbial community associations with switchgrass yields and plant C/N contents

Compared to the control plots, N fertilization of plots at 100 and 200 kg N ha^-1^ increased switchgrass yields by 43% and 171%, respectively (Table 2). In addition, N inputs also significantly increased plant tissue N, but reduced relative C content and C/N ratios (*P* < 0.05) as measured at the end of the growing season. However, soil inorganic N analyses, including NH_3_^+^-N and NO_3_^-^-N, of summer samples showed no difference among the three N fertilization levels (data not shown), indicating that applied N fertilizers were almost completely depleted in soil within approximately four months following N inputs. The DistLM analysis showed that switchgrass yields were significantly correlated with the community structure of bacteria/archaea and fungi, but explained only a small portion of variation, *i*.*e*., 2.6%, 1.2%, in bacterial/archaeal and fungal profiles, respectively (*P* < 0.01) (Table 2), suggesting a small but significant correlation between above-ground switchgrass growth and below-ground microbiomes through N fertilization.

**Table 2:** Above-ground biomass yields and plant C/N contents of switchgrass affected by N fertilization levels as well as their association with community structure of bacteria/archaea and fungi by marginal test of DistLM. Different letters within each column indicate significant effects by N fertilization levels at α = 0.05. The *, **, and *** indicate significant DistLM relationship at α = 0.05, 0.01, and 0.001, respectively.

	Yield (Mg Ha^-1^)	C (%)	N (%)	C/N
N level (kg N ha^-1^)				
0	29.6 b	50.43 a	0.28 a	189.46 a
100	42.4 ab	49.70 b	0.83 b	74.73 b
200	80.1 a	49.47 b	0.98 b	60.62 b
Proportion of explained variation				
Bacteria/archaea	2.6%***	1.2%*	1.0%	0.6%
Fungi	1.2%**	1.0%	1.0%	0.8%

## Discussion

### Short-term N effects on microbial communities

Long-term N inputs, whether through fertilization or atmospheric deposition, can alter microbial composition and diversity through N-induced soil acidification and fertility decline [11]. Many long-term studies have reported that N fertilization not only reduces below-ground biodiversity but also shifts bacterial composition at the phylum level, for groups such as Proteobacteria, Acidobacteria and Actinobacteria [45–49]. Field studies focusing on short-term effects of N fertilization on microbial communities however are limited in number for comparison. In our study, one-time fertilization did not affect the richness and diversity of soil microbial communities, but caused more limited structural changes in both bacterial/archaeal and fungal community composition (Fig 4). Our work suggests that some phylogenetic groups of bacteria and fungi might quickly react either to N inputs directly or through indirect plant associated mechanisms, even when soil properties are not significantly modified by short-term N fertilization. These small but subtle N effects were consistent across the two soil depths and three sampling times after the N inputs, because there was no significant interaction between N and depth/season (Table 1). We also observed that the one-time N amendment appeared to directly repress some bacterial and fungal groups based on the negative relationships of relative abundance with N levels, for example, bacterial genus *Pseudonocardia* and fungal genus *Archaeorhizomyces* (Fig 5). However, we did not observe any changes in organism classifications that might be traditionally associated with ammonia or nitrate transformations (e.g., nitrification or denitrification).

The *Pseudonocardia* is a common endophytic Actinomycete frequently isolated from host plant tissues [50], which has been reported to achieve associative nitrogen fixation without the formation of nodules [51] and protect their hosts against soil-borne pathogenic infection through producing antibiotics or siderophores [52, 53]. As a free living diazotrophic Actinomycete, it has also been reported to be prominent in nutrient limited environments [54, 55] or low-input agro-ecosystems [56, 57], due to its low requirement for N. Based on sequencing of 16S rRNA genes, it was also found that *Pseudonocardia* OTUs were reduced in the fertilized plant rhizospheres of Canola (*Brassica napus*) [58]. Our results support that the relative abundances of *Pseudonocardia* are significantly and negatively associated with N fertilization (Fig 5), and suggest that even short-term N inputs might acutely suppress this associative nitrogen fixer in switchgrass cultivated lands.

The *Archaeorhizomyces* are an ancient class of ubiquitous soil fungi [59], which are neither mycorrhizal nor pathogenic, but may be root endophytic or free-living saprophytes [60]. This group was first discovered in tundra soils [61] using rRNA-based sequencing, but was only isolated into culture more recently [59]. Very little is definitively known about the physiology and ecology of this group and this knowledge comes from only one extant isolate of what is a broad class of organisms. Much of our knowledge of this group accordingly only comes from molecular microbial ecology studies like this one, examining changes in these organisms under different conditions. For example, by investigating how organic matter accumulation and forest fertility influences fungal community composition, it was found from ITS rRNA gene analyses that *Archaeorhizomyces* dominated the root-associated Ascomycetes and their abundance significantly correlated with a fertility gradient in European boreal forests [62]. Moreover, it has been shown that the relative abundance of *Archaeorhizomyces* in grasslands is greatly stimulated by amendment of the biofertilizers *Trichoderma* [63] and correlations between soil properties and fungal abundance suggested that soil P availability (rather than N) may be a controlling factor for *Archaeorhizomyces* relative abundance. However, in our study, inorganic N fertilization significantly reduced the relative abundance of *Archaeorhizomyces*, which was one of the dominant groups of Ascomycota present in our study at 3.1–6.9% relative abundance (Fig 5). Further studies on the ecology of these diverse fungi are clearly needed through both additional rRNA gene amplicon studies in natural systems, as well as the isolation of additional representatives for ecophysiological analyses.

### Spatial heterogeneity in microbial communities

Several studies have reported that soil bacterial and fungal diversity levels can either decrease [25, 64, 65], remain unchanged [27, 66, 67] or increase [68] with soil depth. We consistently observed reduced community richness and diversity in 5–15 cm compared to 0–5 cm soil layers for both bacterial/archaeal and fungal communities (Fig 3). Since plant residue serves as a key carbon source for soil microbes, the vertical distribution of microbial communities is likely to reflect the different available organic matter content with soil depths for microbial decomposers [65]. For example, the surface soil may have more easily decomposable carbon directly derived from crop residues, with more diverse groups of microbes able to access the labile organic materials in this niche [69] whereas subsurface soils may harbor relatively more recalcitrant carbon sources or be more dependent on root inputs. We also observed less soil C and N in 5–15 cm soil layers, further suggesting nutrient levels may be among the factors driving these depth related patterns in diversity.

Compared to the small amount of community variation attributable to N addition, we observed more significant shifts in both bacterial/archaeal and fungal community composition between soil depths. Generally, Bacteroidetes, Planctomycetes, and Verrucomicrobia were more abundant in the 0–5 cm soil layer. This spatial differentiation of the dominant bacterial groups by soil depth was consistent to many previous studies. For example, it has been shown that bacterial community composition was significantly altered at different soil depths, which was associated primarily with a decline of Bacteroidetes with depth [25]. Others have also reported that Verrucomicrobia exhibit higher relative abundance in the surface soils [27, 68]. In contrast, our results showed that the 5–15 cm soil layer had greater abundance in the phyla of Chloroflexi, Nitrospirae, and Proteobacteria. Similarly, it is also demonstrated that as soil depth increased, the relative abundance of Proteobacteria increased and it became the dominant bacterial group in subsoil [66]. Though the overall Proteobacteria were more abundant in 5–15 cm soils, the class Betaproteobacteria was most abundant in 0–5 cm, which was also found in other study [70]. Similar to our results, others have also reported that Chloroflexi [67, 68] and Nitrospirae [71] increase in abundance with soil depth.

In this study, we observed that fungal community also showed strong vertical distribution patterns in the major groups, such as Ascomycota, Chytridiomycota, and Glomeromycota, which were more abundant in top 0–5 cm soil layer; however, compared to bacterial community, there were overall fewer fungal taxonomic groups that differed between soil depths (Figs 6 and 7). Several studies have highlighted the ecological significance of vertically distinct fungal communities. For example, using pyrosequencing of ITS amplicon, others have found a decrease in relative abundance of Ascomycota with increasing soil depth, whereas Zygomycota showed the opposite trend [64]. At finer taxonomic scales, it was reported that Sordariomycetes of the phylum Ascomycota decrease with soil depth [71], and this pattern was similar in our study. Others have also shown overall fungal communities pattern were highly variable with soil depth, where deeper soil have some distinct fungal groups, but significantly less overall diversity [65] similar to what we observe here.

### Temporal variation in microbial communities

We found significant temporal changes in alpha diversity in both bacterial and fungal communities (Fig 3). Although the evaluation of chronosequences of microbial community has been of interest to study in switchgrass cultivation [72], our results showed a significant temporal variation of both bacterial and fungal communities even in one year. Considering such variation is important as these significant temporal effects may override the changes from N additions. Only by applying a well replicated design and accounting for temporal and depth variation in our study, could we thoroughly assess the differences between the N-free controls and N fertilization treatments. It has previously been shown that bacterial community alpha diversity varies more substantially than beta diversity over time, and in this case exceeded the variability between land-use types [26], which attributed to rhizodeposition as a controlling factor on soil bacterial diversity. Our data show that seasonal variation is found in most dominant phylogenetic groups of bacteria, such as Acidobacteria, Actinobacteria, Bacteroidetes, Chloroflexi, and Verrucomicrobia (Fig 6). Interestingly Acidobacteria, Bacteroidetes, Betaproteobacteria, Deltaproteobacteria, Gammaproteobacteria, and Verrucomicrobia all had similar seasonal patterns, which were opposite to those of Actinobacteria, Chloroflexi, and Alphaproteobacteria. That dominant bacterial phyla, such as Actinobacteria and Betaproteobacteria, shift seasonally to temporal patterns has also been reported previously [66]. In addition, it has been shown that seasonal dynamics often appear to be coherent within taxonomic lineages, in which Acidobacteria and Proteobacteria are more prevalent in summer, whereas Actinobacteria and Chloroflexi increase in winter [73].

In our experiment, the fungal phyla Chytridiomycota, Glomeromycota, and Zygomycota, were also found to vary significantly over the different seasonal sampling dates (Fig 7). It has been reported that Ascomycota and Glomeromycota increase in summer, whereas Basidiomycota are dominant in winter [74] or alternatively that Ascomycota, Basidiomycota, and Zygomycota are variable from spring to winter [75]. Changes in litter decomposition and phytosynthate allocation likely also contribute to the seasonal variations of fungal community [76] in addition to the direct effects of soil moisture and temperature [26]. However, while season is important in overall community structure, contrary to the above, we did not observe distinct seasonal changes in the overall dominant patterns of Ascomycota or Basidiomycota in our study and in any case such patterns are likely to be ecosystem specific.

## Conclusions

With the aid of high-throughput 16S rRNA gene and ITS region amplicon sequencing, we found highly diverse and dynamic communities across this 8 year-old switchgrass field. The one-time application of N fertilization significantly stimulated switchgrass growth and N uptake, and subtly but significantly shifted below-ground bacterial and fungal communities, with the bacterial genus *Pseudonocardia* and *Archaeorhizomyces* fungi negatively responsive to N inputs. However, these shifts took place within the context of much larger spatial and temporal variation in the microbial community. Only by using a highly replicated study, and thoroughly accounting large spatial and seasonal fluctuations in microbial communities, could we account for these subtle differences. This reinforces the importance of robust sampling designs and should caution against overinterpretation of studies based on one-time sampling events. Follow-up studies should aim at obtaining a better understanding of the ecological and physiological mechanisms of responses to N fertilization by these microbes and how these may influence ecosystem functions.

## Supporting information

S1 Fig Relative abundances of soil metagenomes annotated in RefSeq database (RefSeq), and functional classification annotated in SEED Subsystems database (SEED Subsystems) in composite soils sampled at two soil depths (0–5 and 5–15 cm) in Winter 2016. Asterisks indicate significant difference at α = 0.05 between two soil depths.
(TIF)

S2 Fig Non-metric multidimensional scaling (NMDS) analysis of 24 putative functions at KEGG level 1 predicted by PICRUSt based on 16S rRNA amplicons compared with 4 soil metagenomes annotated in KO database (KEGG level 1) using soils sampled in Winter 2016. PERMANOVA *P* values were also given.
(TIF)

S3 Fig A linear discriminant analysis effect size (LEfSe) method showing dominant functional groups (average abundances > 1%) that were significantly different between soil metagenomes annotated in KO database by MG-RAST and putative functions in KEGG predicted by PICRUSt based on 16S rRNA amplicons using soils sampled in Winter 2016. From the center outward, each circle represents the KEGG level 1, 2, and 3, respectively. The functional groups with significant differences are labeled by red color (MG-RAST) or green colors (PICRUSt).
(TIF)
