# Supplementary figures and images for: One-time nitrogen fertilization shifts switchgrass soil microbiomes within a context of larger spatial and temporal variation
Source: PLoS One. 2019 Jun 18;14(6):e0211310. doi: 10.1371/journal.pone.0211310 (PMC6581249; doi:10.1371/journal.pone.0211310)

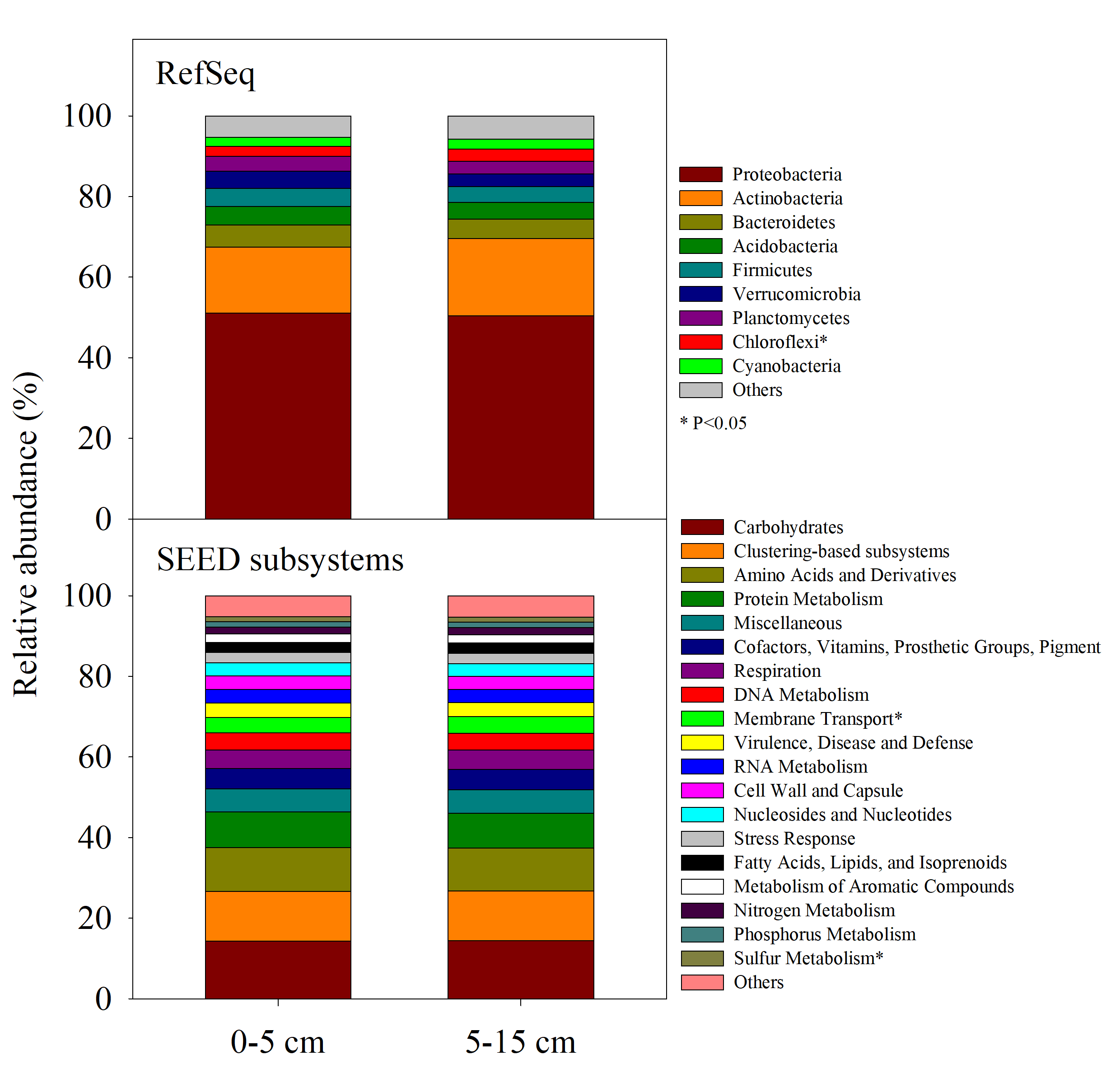

Supplement: S1 Fig — Asterisks indicate significant difference at α = 0.05 between two soil depths. (TIF) [file pone.0211310.s001.TIF]

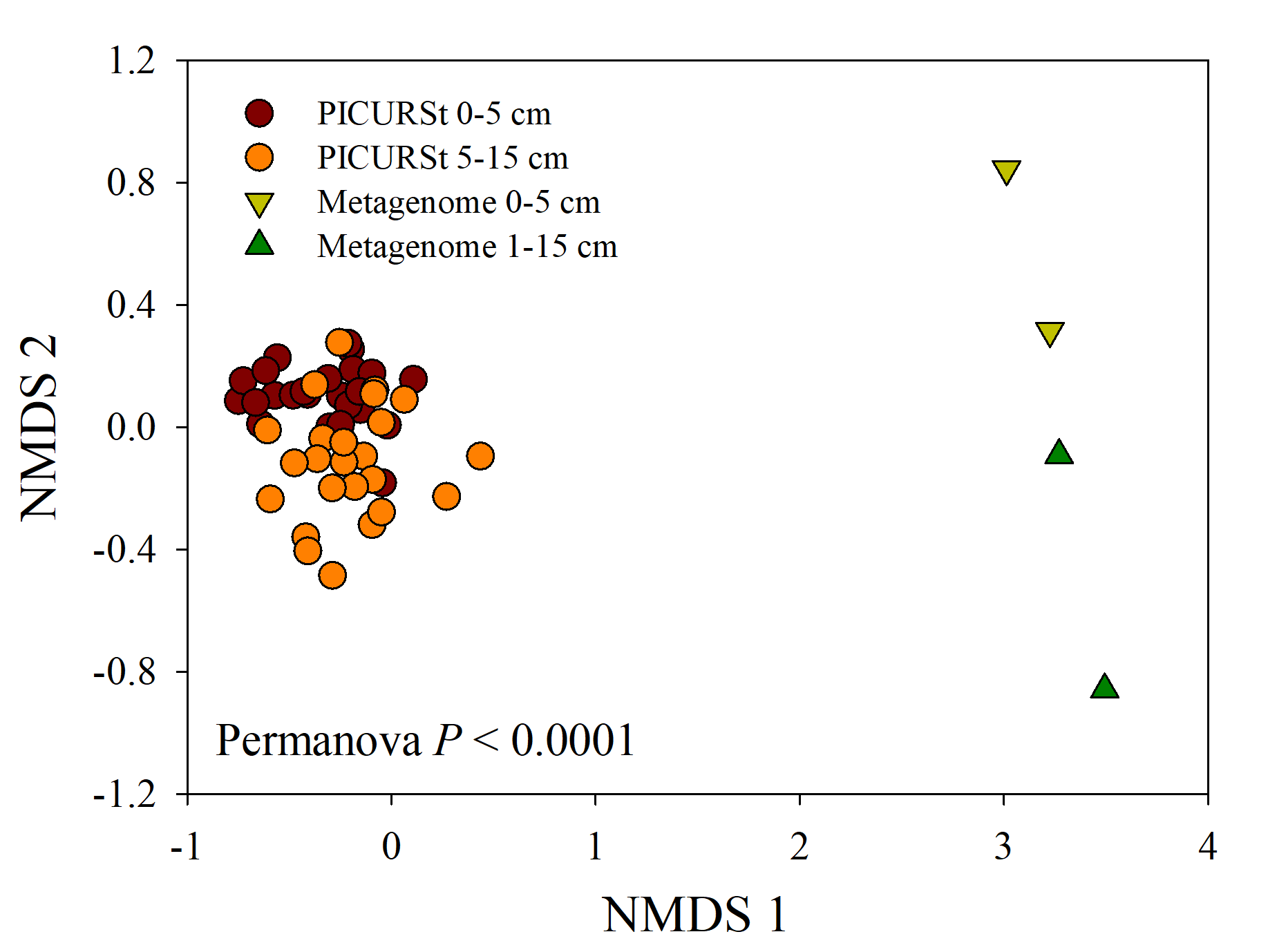

Supplement: S2 Fig — PERMANOVA P values were also given. (TIF) [file pone.0211310.s002.TIF]

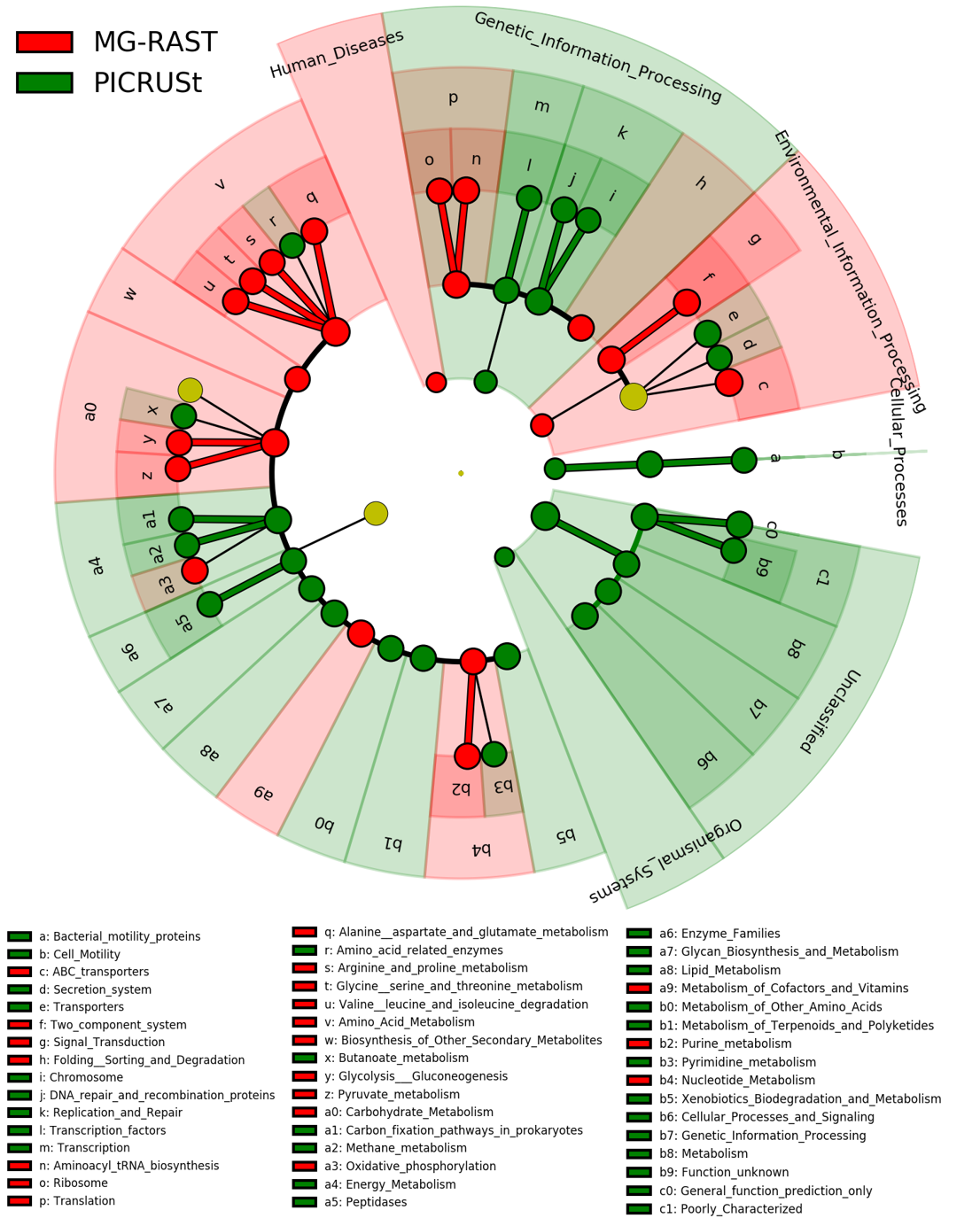

Supplement: S3 Fig — From the center outward, each circle represents the KEGG level 1, 2, and 3, respectively. The functional groups with significant differences are labeled by red color (MG-RAST) or green colors (PICRUSt). (TIF) [file pone.0211310.s003.tif]
